# Underexpression of miR-126-3p in Patients with Cholangiocarcinoma

**DOI:** 10.31557/APJCP.2021.22.2.573

**Published:** 2021-02

**Authors:** Lucas P Spinola, Gabriel F Vieira, Rafael Fernandes-Ferreira, Maria CJ Calastri, Graciele D Tenani, Franciana L Aguiar, Ilka FSF Boin, Larissa BE Costa, Maria Fernanda C Correia, Eliane M Zanovelo, Daniele CB Souza, Rita CMA Silva, Renato F Silva, Ana Margarida C Abrantes, Maria Filomena R R Botelho, José Guilherme L R Tralhão, Dorotéia RS Souza

**Affiliations:** 1 *Department of Molecular Biology, São José do Rio Preto Medical School (FAMERP), São Paulo, Brazil. *; 2 *School of Medical Sciences of the State University of Campinas (UNICAMP), Campinas, Brazil. *; 3 *Base Hospital (HB), São José do Rio Preto, São Paulo, Brazil. *; 4 *Medical School of the University of Coimbra, Coimbra, Portugal. *

**Keywords:** Liver Cancer, microRNA, angiogenesis, lifestyle, comorbidities, survival

## Abstract

**Objectives:**

To evaluate the expression of miR-126-3p and its potential as a biomarker for cholangiocarcinoma (CCA) and to better understand the prognosis, comorbidities, and lifestyle habits associated with the disease.

**Methods:**

Fifty-nine individuals were distributed into either the study group (38 CCA patients) or the control group (21 individuals without liver diseases). Total RNA was extracted, cDNA synthesis was performed, and miR-126-3p expression was assessed using real-time PCR. For statistical analysis, alpha error was set at 5%.

**Results:**

MiR-126-3p was found to be underexpressed in the study group relative to the controls (0.42; P=0.001). Additionally, marked underexpression was found in the study group in when associated with smoking (0.28; P=0.0001), alcoholism (0.19; P=0.0001), hypertension (0.29; P=000.1), and diabetes (0.12; P=0.0003) relative to the controls. No association was found between miR-126-3p expression and tumor subtypes (iCCA=0.42; pCCA=0.45; dCCA=0.72; P=0.9155). A total of 67% of dCCA patients were event-free at 16 months of follow up, while both pCCA and iCCA exhibited event-free survival rates of 25%, though there was no significant difference between these subgroups (P=0.273).

**Conclusion:**

The underexpression of mir-126-3p is associated with cholangiocarcinoma and can be potentiated by alcoholism, hypertension, diabetes, and smoking, the latter of which is an independent risk factor for this cancer. Furthermore, dCCA patients exhibit higher survival rates relative to patients with pCCA and iCCA.

## Introduction

Cholangiocarcinoma (CCA) is the second most common primary liver cancer after hepatocellular carcinoma (HCC), accounting for 10% to 15% of primary liver malignancy. CCA is formed in the epithelial tissue that makes up the biliary tract and is classified into three anatomical subtypes: intrahepatic carcinoma (iCCA), perihilar carcinoma (pCCA), and distal carcinoma (dCCA) (Khan et al., 2019; Massironi et al., 2020).

Viral infections, primary sclerosing cholangitis, biliary lithiasis, congenital malformations, cirrhosis of the liver, and hepatobiliary parasite infections have been found to be risk factors for CCA (Khan et al., 2019). Lifestyle habits, such as alcoholism and smoking, as well as comorbidities, namely diabetes mellitus (DM), also contribute to the risk of CCA (Brandi et al., 2020).

MicroRNAs (miRNAs), which are small fragments of non-coding RNA containing approximately 22 nucleotides, are known to play a role in the complex process that is carcinogenesis. Several studies have determined an association between differing miRNA expression and the development of malignant tumors, suggesting the potential of miRNAs in the use of cancer diagnoses, prognoses, and treatments (Khan et al., 2019; Jiang et al., 2020).

MiR-126, which is located in intron 7 of the EGFL7 (epidermal growth factor) gene, encodes a factor derived from the endothelial cells and involved in suppressing smooth muscle cell migration and angiogenesis regulation (Parker et al., 2004). MiR-126 is represented as miR-126-5p and miR-126-3p, which correspond to the 5’and 3’ regions of the transcription, respectively (Hu et al., 2019).

MiR-126-3p acts as a tumor suppressor by negatively regulating some genes, such as the gene that codes for vascular endothelial growth factor (VEGF-A) and solute transporter family 7 member 5 (SLC7A5). Thus, lower expression of miR-126-3p facilitates tumor cell formation, migration, and invasion, and contributes to angiogenesis progression (Zhang et al., 2013; Caporali et al., 2019; Sabryet al., 2019). It is well established that miR-126-3p is underexpressed in certain types of cancer, including cancers of the esophagus and the pancreas, as well as in glioma and HCC (Atansov et al., 2018; Lou et al., 2018; Luo et al., 2019; Wu et al., 2019). Therefore, this study sought to evaluate the expression of miR-126-3p and its potential as a biomarker for CCA, in addition to CCA prognosis and the comorbidities and lifestyle habits associated with the disease.

## Materials and Methods

*Patients*

Fifty-nine individuals who varied in terms of sex, ethnicity, and age were included in the study and distributed into either the study group (38 CCA patients whose tumor samples had been collected and stored in paraffin blocks) or the control group, which consisted of 21 individuals with no clinical signs of any liver diseases.

All study group patients were selected after a histopathological assessment performed at the Clinical Pathology Department of Base Hospital, the teaching hospital affiliated with the São José do Rio Preto Medical School (HB/FAMERP). Patients’ clinical, demographic, and lifestyle habits data were obtained from electronic medical records and questionnaires. The controls were selected at the Liver Transplantation Department of HB/FAMERP after having provided cystic duct samples during prior laparoscopic cholecystectomy procedures. All individuals were informed of the details of the study. The controls agreed to participate by signing the informed consent form. Because the experimental samples were obtained from the local tumor bank, study group patient inclusion approval was obtained from the FAMERP Research Ethics Committee.

*Analysis of miR-126 Expression*

Total RNA was extracted from formalin-fixed, paraffin-embedded (FFPE) blocks using the ReliaPrep™ FFPE Total RNA Miniprep System kit (Promega) according to the manufacturer’s instructions. The material collected from the controls was immediately stored in RNAlater for RNA extraction using the TRIzol reagent according to the manufacturer’s instructions. Next, concentration and purity ratios were measured on the NanoDrop (NanoDrop Technologies, Inc., Wilmington, DE) considering the absorbance of the sample (ratio of 260:280 and 1.7–2.0, respectively).

To perform the reverse transcription of miR-126-3p, the TaqMan MicroRNA Reverse Transcription kit was used with a specific assay for miR-126-3 (assay: 002228, Applied Biosystems, Foster City, CA, USA). The reaction was placed in a thermocycler with cycling conditions of 16°C for 30 minutes, 42°C for 30 minutes, 85°C for 5 minutes and 4°C - hold ∞.

Real-time polymerase chain reaction (RT-qPCR) was performed on the cDNA samples for to analyze the relative expression of miR-126-3p. The thermal cycling conditions used were those recommended by the StepOne Plus device (Applied Biosystems). All analyses were carried out in triplicate.

*Analysis of the Results*

Statistical analyses were performed using the IBM® SPSS® Statistics software, version 20.0 (IBM Corporation, Armonk, New York, USA), StatsDirect, and GraphPad. The descriptive analysis of the variables and the results have been presented as median, minimum, and maximum. Fisher’s exact test analyzed the frequency distribution of the patients’ clinical-demographic profiles, lifestyle habits, and comorbidities. In the case of the non-parametric quantitative variables, the Kruskal-Wallis test was used to compare three or more groups; the Mann-Whitney test was applied to two groups. The relative expression of miR-126-3p was calculated using the 2^-∆∆Ct ^method, and the reaction was normalized by the housekeeping genes U6 and RNU 48. A receiver operating characteristic (ROC) curve was used to evaluate the discriminatory power of the molecular markers and to determine the cut-off, sensitivity, and specificity values, considering areas under the curve greater than or equal to 0.7 as clinically relevant. Patient survival was assessed using the Kaplan-Meier survival curve. An alpha error of 5% was set for intergroup comparison.

## Results

The demographic profile analysis (Table 1) revealed a higher median age range in the study group (median=58 years) than in the control group (median=39 years; P=0.0011), with a prevalence of females in both groups (study group=55%; control group=81%; P=0.0870). The subjects were similar to other members of their groups in terms of lifestyle habits and comorbidities (P>0.05, Table 1), particularly in the case of smoking among CCA patients (29%; control group=10%), in rates of alcoholism and DM among the controls (24% and 19%, respectively; study group = 21% and 11%, respectively), and in the rate of systemic arterial hypertension, or SAH (24% in both groups). A higher rate of the intrahepatic tumor subtype (iCCA; 47%) was observed, followed by the rate of perihilar CCA, or pCCA (42%) and distal CCA, or dCCA (11%; Table 1).

**Figure 1:**
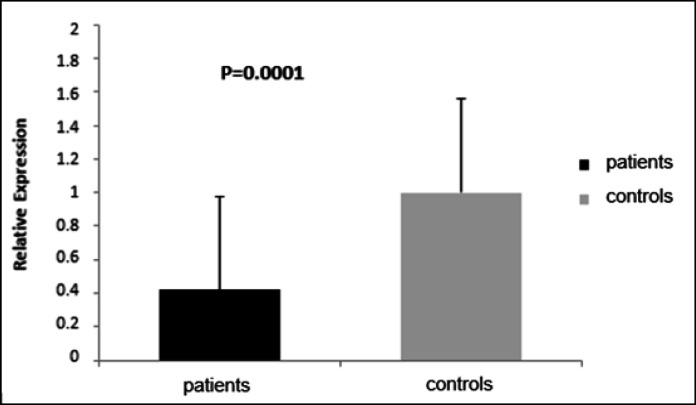
Relative Expression of miR-126-3p (mean value of 2-ddCt) in Patients with Cholangiocarcinoma (Patients) and Individuals without the Disease (controls). Significance level=P<0.05

**Figure 2:**
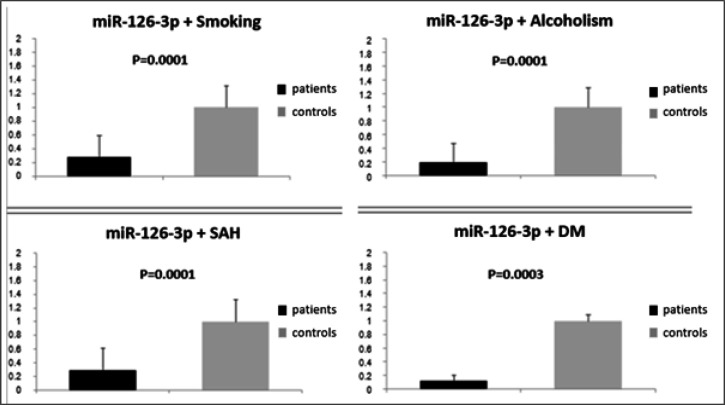
Relative Expression of miR-126-3p (mean value of 2-ddCt) Associated with Lifestyle Habits (Smoking and Alcoholism) and Comorbidities (Systemic Arterial Hypertension [SAH] and Diabetes Mellitus [DM]) in Patients with Cholangiocarcinoma (Patients) and Individuals without the Disease (Controls). Significance level=P<0.05

The study group exhibited underexpression of miR-126-3p relative to the control group (0.42; P=0.001; Figure 1). Additionally, underexpression was noteworthy in the study group in the presence of smoking (0.28; P=0.0001), alcoholism (0.19; P=0.0001), SAH (0.29; P=000.1), and DM (0.12; P=0.0003) relative to the controls (Figure 2). No association was found between miR-126-3p expression and tumor subtypes (iCCA=0.42; pCCA=0.45; dCCA=0.72; P=0.9155).

MiR-126-3p expression was also analyzed in the context of patient pathology. There was a similarity between patients with (53%) and without metastasis; and yet with more marked underexpression in patients with metastasis (0.34 versus 0.47, respectively; P=0.5523).

**Figure 3:**
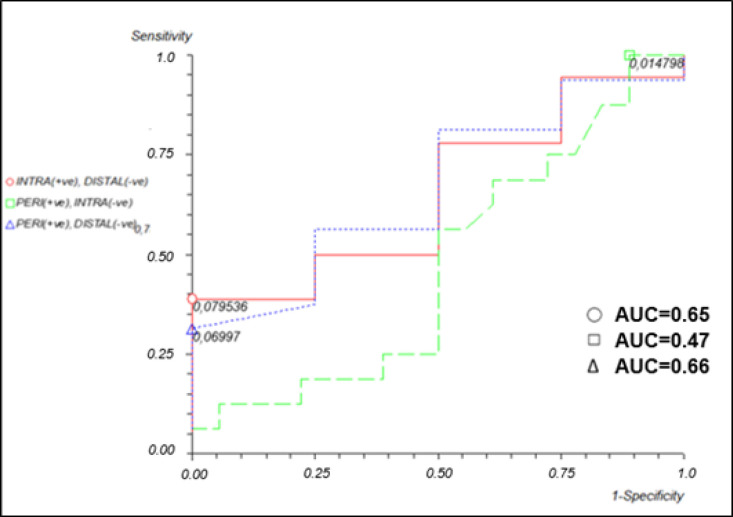
Receiver Operating Characteristic (ROC) Curve of the Levels of Relative Expression of miR-126-3p in Patients with Intrahepatic, Perihilar, or Distal Cholangiocarcinoma (CCA).

The discriminatory power of miR-126-3p in relation to tumor subtype was also assessed (Figure 3), though the analysis revealed no significant difference in expression between iCCA patients and dCCA patients (area under the curve [AUC]=0.65, 38% sensitivity, and 100% specificity), between pCCA patients and iCCA patients (AUC=0.47, 100% sensitivity, and11% specificity), or between pCCA patients and dCCA patients (AUC=0.66, 31% sensitivity, and 100% specificity).

**Figure 4.**
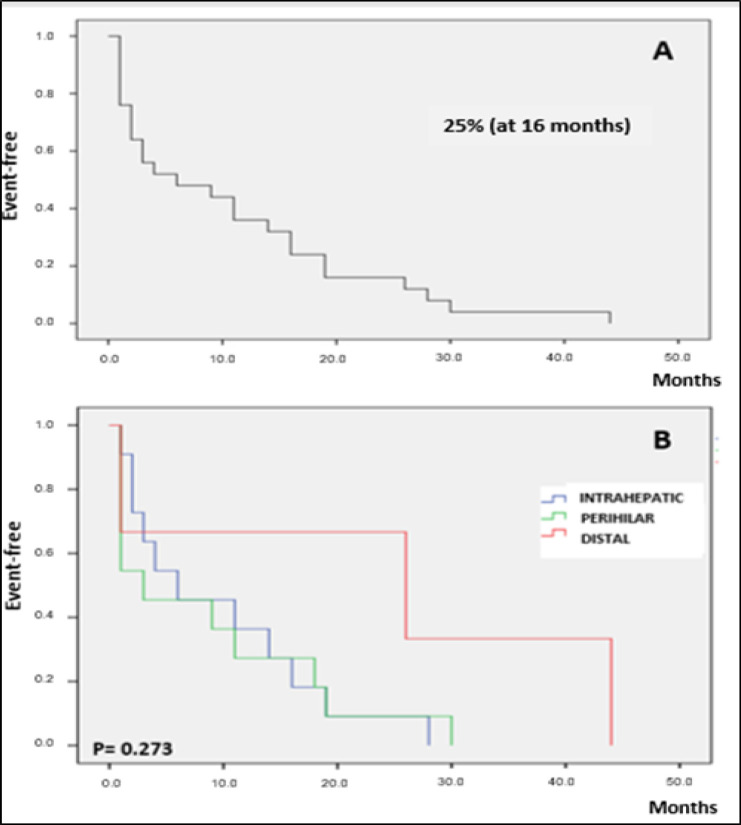
Kaplan-Meier Curve for Analysis of Overall Survival Rates of Patients with Cholangiocarcinoma (A) and Survival Rates Separated by the Intrahepatic, Perihilar, and Distal Tumor Subtypes (B) at Follow up up to 44 Months after Diagnosis

CCA patient survival was assessed using the Kaplan-Meier survival curve as of the date of disease diagnosis. The median survival time for the three tumor subtypes was 16 months. The rate of event-free survival among the study group as a whole was 25% at 16 months (Figure 4A), with similar results regardless of tumor subtype (P>0.05; Figure 4B). However, the event-free survival rate among dCCA patients was 67% at 16 months. This rate was markedly (but not significantly) higher than the 25% rate found for both pCCA and iCCA patients (P=0.273).

The logistic regression analysis to identify independent variables and a given individual’s risk of developing CCA produced the following equation: logit Y=0.435021 +2.137175 smoking - 1.07963 alcoholism + 0.687901 HAS - 1.191965 DM. It also revealed smoking to be an independent factor for CCA (P=0.039; Table 2), unlike alcoholism, SAH, or DM.

**Table 1:** Demographic Profile, Lifestyle Habits, Comorbidities, and Clinical Classification of Patients with Cholangiocarcinoma (Patients) and Individuals without the Disease (Controls)

	Patients (N= 38)		Controls (N= 21)		Patients vs. Controls
Age (years)					*P
Median	58		39		
Minimum	30		24		0.0011
Maximum	79		67		
Sex	N	%	N	%	*P
Male	17	45	4	19	0.087
Female	21	55	17	81	
Lifestyle Habits	N	%	N	%	
Smoking	11	29	2	10	0.1092
Alcoholism	8	21	5	24	1
Comorbidities	N	%	N	%	
DM	4	11	4	19	0.4375
SAH	9	24	5	24	1
Clinical Classification	SG (N=38)				
Tumor Subtype	N		%		
Intrahepatic	18		47		
Perihilar	16		42		
Distal	4		11		

Significance level=P<0.05/Mann-Whitney test and Fisher’s test; N, Number of individuals; DM, diabetes mellitus; SAH, systemic arterial hypertension

**Table 2:** Cholangiocarcinoma Patient versus Control Group Risk Profile as Determined by Logistic Regression Analysis

	Patients vs Controls		
Covariant	Odds Ratio	Confidence Interval	P
Smoking	8.47	1.11-64.47	0.039
Alcoholism	0.33	0.05-2.18	0.2559
SAH	1.98	0.38-10.33	0.4133
DM	0.30	0.03-2.49	0.2673

SAH, systemic arterial hypertension; DM, diabetes mellitus; significance level=P<0.05

## Discussion

In this study of Brazilian patients, females were the majority in the CCA patient group, a rate which is in line with studies on Hispanic and Thai populations (1.5 and 0.9 per 100,000 inhabitants and 55.6%, respectively) (Everhart and Ruhl, 2009; Khuntikeo et al., 2015). It is important to note that estrogen, the main hormone responsible for secondary sexual characteristics in females, regulates the growth of hyperplastic and neoplastic cholangiocytes (Alvaro et al., 2007). Alvaro et al., (2004), Alvaro et al., (2006) found estrogen receptor alpha (RE-α) expression to be four times higher in malignant cholangiocytes than in cholangiocytes in cases of benign diseases, such as primary sclerosing cholangitis, primary biliary cirrhosis, and alcoholic cirrhosis. Conversely, other studies have reported a higher incidence of CCA in males (Shaib et al., 2004; Tyson et al., 2011; Rizvi et al., 2013). In any case, more information is needed on the molecular foundations of sex differences in CCA (Rubin, 2020).

A larger age range was also observed in patients with CCA relative to controls, a finding in agreement with Khan (2019), who demonstrated that this type of tumor is uncommon in individuals under 40 years of age. Although cancer also affects young people, the incidence and mortality of most types of cancer increase exponentially with age (Yanciket al., 2004). DNA is continually damaged by mutagenic agents of intrinsic and extrinsic origins during human growth and aging. Most of this damage is repaired, but a small fraction can become permanent mutations. Inefficiency in DNA repair, the gradual accumulation of mutations, and epigenetic alterations are processes associated with aging that impair the integrity and stability of the genome and favor the malignant transformation of cells (Greenmanet al., 2007; Stratton et al., 2009; Vjiget al., 2014).

In our study, smoking was found to be an independent risk factor for CCA, a result which is in agreement with the literature (Ye et al., 2013). Petrick et al., (2017) demonstrated that smoking accounts for 46% of iCCA risk and 77% of extrahepatic CCA risk. It is well known that smoking is associated with several types of cancers; in CCA, this association results from the presence of cancer-causing compounds in tobacco that can damage the biliary epithelium through its direct exposure to the bloodstream. Tobacco has more than 40 active compounds metabolized by the liver, including polycyclic aromatic hydrocarbons, formaldehyde, benzene, chromium, nitrosamines, and aromatic amines, which are responsible for DNA damage, a key triggering event for carcinogenesis (Ye et al., 2013; Petrick et al., 2017; Peder LD and Franke, 2019).

Although alcoholism was found to be associated with similar risks between the subtypes, a three-fold higher risk for iCCA has been found to be associated with chronic alcoholism (Palmer et al., 2012). In this case, there is a relationship between carcinogenesis, increased production of reactive oxygen species (ROS), and the antioxidant deficiency that results from the oxidation of ethanol, processes which interfere with DNA synthesis and repair (Petrick et al., 2018; Dumitrescu, 2018).

In this study, the patients and controls were found to have similar rates of DM. However, this comorbidity is an important factor in carcinogenesis (Shafqet et al., 2017) in that hyperinsulinemia stimulates greater expression of type 1 insulin-like growth factor (IGF-1) and insulin receptors via PI3K/Akt/mTOR signaling, thus triggering proliferation, angiogenesis, metastasis, and inhibition of apoptosis (Wojciechowskaet al., 2016; Labibet al., 2019; Clements et al., 2020). It is important to note that our sample contained a majority of women; however, the global incidence of DM among men has risen in recent decades (NCD Risk Factor Collaboration, 2016).

Hypertension was not found to be associated with CCA, results that were in line with a recent study (Clements et al., 2020). Nevertheless, a relationship between this comorbidity and the development of cancer has been suggested in another study. In this case, carcinogenesis was found to be relevantly associated with SAH and the hyperexpression of VEGF and angiotensin II (namely, important angiogenic factors), since carcinogenesis induces the formation of new blood vessels and thus enables tumor growth (Tiniet al., 2019).

The iCCA and pCCA subtypes were found to be the most frequent in this study population; in other studies, the pCCA subtype prevailed (Mansour et al., 2015; Waseem et al., 2017). It is important to note that the number of cases of iCCA have been increasing, particularly in Western countries (Mc Lean et al., 2006; Razumilavaet al., 2014), a change which may be attributed to the higher frequency of risk factors for this tumor subtype (Sigalet al., 2012; Massarwehet al., 2017).

In the present study, miR-126-3p expression was lower in the tumor tissue obtained from patients with CCA, as has been observed in other studies (McNally et al., 2013; Atanasovet al., 2018). MiRNAs act as post-transcriptional regulators by combining the 3’untranslated (3’UTR) regions of the target RNAs (mRNA) of the target genes, causing mRNA degradation or translation suppression. In this context, the underexpression of miR-126-3p can positively regulate VEGF, one of the target genes of miR-126-3p, and thus contribute to tumor hypervascularization, which meets the tumor’s need for a blood supply, nutrient transport, oxygen, and waste removal for tumor viability and cell proliferation (Shim et al., 2008; Ratnasariet al., 2016; Hong et al., 2018). It is also important to note that miR-126-3p is markedly underexpressed in patients with CCA in this population, particularly when associated with smoking, alcoholism, SAH, and DM, all of which can influence disease progression and prognosis. These associations should be investigated further in prospective studies.

Overall CCA patient survival was 25% at 16 months of follow up; in the dCCA subgroup, however, 67% of patients were event-free at this point, a rate which is consistent with those of other studies (Waseem andTushar, 2017). There is also evidence that patients with dCCA respond better to surgical treatment, especially when it is combined with other treatment options, such as chemotherapy (Waseem andTushar, 2017).

Metastasis was observed in 53% of patients with CCA. A recent study reported that most patients with iCCA exhibit metastasis, and that liver metastases are particularly common due to the hypervascularization of this organ (Yan et al., 2019). Another study found metastasis in 44% of CCA patients (Ma et al., 2015). On the other hand, Zhao (2014) observed metastasis in only 11% of patients with iCCA; while Nojiet al. (2012) recorded 60% and 48% of lymph node metastases in pCCA and dCCA patients, respectively. In CCA, the underexpression of miR-126-3p intensifies the expression of the EGFL7 gene, which in turn, activates signaling pathways and stimulates cell survival and proliferation, processes which can favor metastasis development (Hong et al., 2018).

In this study, the underexpression of miR-126-3p, a post-transcriptional regulator of genes involved in angiogenesis (VEFG) and cell proliferation (SLC7A5), was found to be associated with CCA and may be potentiated by alcoholism, SAH, DM, and smoking; smoking was also found to be an independent risk factor for CCA. Furthermore, patients with dCCA, a tumor located in the distal extrahepatic bile duct, have a better prognosis in terms of survival when compared to patients with pCCA and iCCA.

## Financing Statement

This research has received financial support from National Council for Scientific and Technological Development (CNPq; Grants 400988/2016-0 and 301704/2017-1) and Coordination for the Improvement of Higher Education Personnel (CAPES).
